# Risk assessment of gastric cancer associated with asbestosis: a case report

**DOI:** 10.1186/s40557-015-0061-4

**Published:** 2015-03-13

**Authors:** Soo-Hong Park, Dong-Mug Kang, Bon-Hak Koo, Young-Ki Kim, Jong-Eun Kim

**Affiliations:** Department of Occupational and Environmental Medicine, Pusan National University Yangsan Hospital, Yangsan, South Korea; Department of Preventive and Occupational Medicine, School of Medicine, Pusan National University, Pusan, South Korea; Environmental Health Center of Asbestos, Pusan National University Yangsan Hospital, Yangsan, South Korea

**Keywords:** Gastric cancer, Asbestosis, Asbestos, Textile, Spinning, Risk assessment

## Abstract

**Background:**

The International Agency for Research on Cancer classifies asbestos as belonging to Carcinogen Group 2A for gastric cancer. We herein report a case of gastric cancer associated with asbestosis and describe the work-related and risk assessments of asbestos exposure for gastric cancer.

**Case presentation:**

The 66-year-old male patient in our case worked in asbestos spinning factories. His level of cumulated asbestos fiber exposure was estimated to be 38.0–71.0 f-yr/cc. Thus, the Excess Life Cancer Risk for lung cancer associated with asbestos exposure was 9,648×10^−5^, almost 9,600 times the value recommended by the United States of America Environmental Protection Agency (1 × 10^−5^). The relative risk of developing lung cancer for this patient was more than 25 f-yr/cc, a well-known criterion for doubling the risk of lung cancer.

**Conclusion:**

The patient’s exposure to high-dose asbestos was sufficient to increase his risk of gastric cancer because as the risk of lung cancer increased, the risk of gastric cancer was due to increase as well. Therefore, occupational asbestos fiber exposure might be associated with gastric cancer in this case.

## Background

According to the International Agency for Research on Cancer (IARC), asbestos is carcinogenic to humans, and exposure to asbestos results in lung cancer [1], mesothelioma [2], ovarian cancer [3], and laryngeal cancer [4] with sufficient evidence in humans. It may also lead to gastric [5], colorectal [6], or pharyngeal cancer [7] with more limited evidence. Furthermore, asbestos exposure can cause other benign conditions such as asbestosis, emphysema, pleural plaque, pleural effusion, and diffuse pleural thickening [8-10].

The IARC suggests that asbestos, an inorganic lead compound, and ingested nitrate or nitrite [11] are harmful occupational and environmental factors for gastric cancer, albeit with limited evidence. However, other general factors are thought to be of more significance to the clinical development of gastric cancer. The disease is one of the most common types of malignancy in Korea. According to reports from the Korean Cancer Registration Statistics Program, gastric cancer accounted for 14.5% of all cancer cases in the country in 2011, second only to thyroid cancer [12].

Histories of intestinal metaplasia [13], gastric ulcer [14], gastric surgery [15] and atrophic gastritis [16] are reportedly common among gastric cancer patients, and their association with the malignancy has been investigated. In previous studies, the association of lifestyle factors such as smoking [17] and alcohol consumption [18] with gastric cancer has been reported. Moreover, studies on carcinogens in food are currently pursued. Most of these investigations indicate that nitrite is one of the food-related carcinogens that might associate with gastric cancer [19]. The IARC recently suggested with sufficient evidence that *Helicobacter pylori (H. pylori)* is carcinogenic to humans, particularly causing gastric cancer [20].

Although asbestos has been suggested to cause gastric cancer, its association with the disease remains unclear, possibly owing to the complication of many other risk factors involved. Cases of multiple asbestos-related malignancies, including gastric cancer, have been reported in Japan [21,22]. Moreover, several cohort studies have been conducted to evaluate the relationship between gastric cancer and asbestos fiber exposure or the mortality rate among workers exposed to asbestos [23-25]. However, there are no reports on gastric cancer with specific risk assessment for asbestos fiber exposure, and it is difficult to apply epidemiologic study results to individual evaluation of work-related risks. Thus, the need for occupational and environmental medicine (OEM) physicians to determine the association of asbestos fiber exposure, among other factors, to gastric cancer in patients with exposure to various risk factors remains challenging. In the resent report, we describe a case of asbestosis, a benign condition caused by exposure to asbestos and concurrent gastric cancer. To the best of our knowledge, this is the first of such cases being reported in the literature. Since evaluating the association between asbestos fiber exposure and gastric cancer by OEM physicians might be of clinical significance, we also performed work-related and risk assessments of asbestos exposure for gastric cancer.

## Case presentation

### Patient

The patient in our case was a 66-year-old man.

### Chief complaint

His chief complaint was the abnormality observed in his chest computed tomography (CT) images.

### Present illness

The patient was initially suspected of having an ulcerative invasive tumor in his stomach based on endoscopic findings by a clinic in Pusan on September 8, 2012. He then underwent total gastrectomy for gastric cancer at Pusan National University Yangsan Hospital on October 2, 2012, followed by six cycles of chemotherapy between January 27, and May 28, 2013. After a chest CT scan on November 11, 2013, he was diagnosed with asbestosis and transferred to the Department of OEM for further evaluation of the association between asbestosis and gastric cancer in his case.

### Medical and social history

The patient in this case had a history of hypertension. He reported smoking 23-packs-per-year until 1995 and consuming one bottle of Soju (alcohol) per week prior to his gastric cancer surgery.

### Occupational history

The patient worked in a textile factory with no asbestos exposure for 18 years (from 1966 to 1983). He later performed asbestos spinning and weaving work for 11 years (from 1983 to 1993) using chrysotile at Masan Gwangsum (1983 – 1984), Seongjin Chemical (1985 – 1992), and in Taehwa Capaxeal (1993).

### Work details and working environment

His asbestos-spinning job involved mixing chrysotile with synthetic fiber during a 12-hour shift (from 8 a.m. to 8 p.m.).The job site was full of white dust, and despite wearing a mask, he still found dust around his nose when finishing work. Between 1987 and1990, as a plant manager, he managed the entire process that involved mixing asbestos and synthetic fiber, spinning, twisting, and weaving. Additionally, he directly spun asbestos.

### Clinical observations

The patient looked healthy at presentation. His blood pressure was 130/70 mmHg; body temperature was 36.6°C; pulse rate was 80 beats/minute, and respiratory rate was 20 times/minute. His conjunctiva was not anemic, and anicteric sclera was observed. His chest auscultation revealed fine crackles of end inspiration in the lower areas of both lungs. His heartbeat was regular, and no heart murmur was detected. The physical examinations showed no abnormal findings in his abdomen or skin, and no club fingers or cyanosis in his extremities.

### Laboratory findings

In a pulmonary function test conducted on November 18, 2013, the patient’s forced expiratory volume at timed intervals of a second (FEV1) was 2.24 L (72% of the expected value); his forced volume vital capacity (FVC) was 2.52 L (61% of the expected value), and his FEV1/FVC was 89% of the expected value, which showed a mildly restrictive ventilator disturbance. His pulmonary diffusion capacity of carbon monoxide (DLco) was within the normal range at 15.9 ml/min/mmHg (77% of the expected value).

### Imaging findings

**Figure 1:**
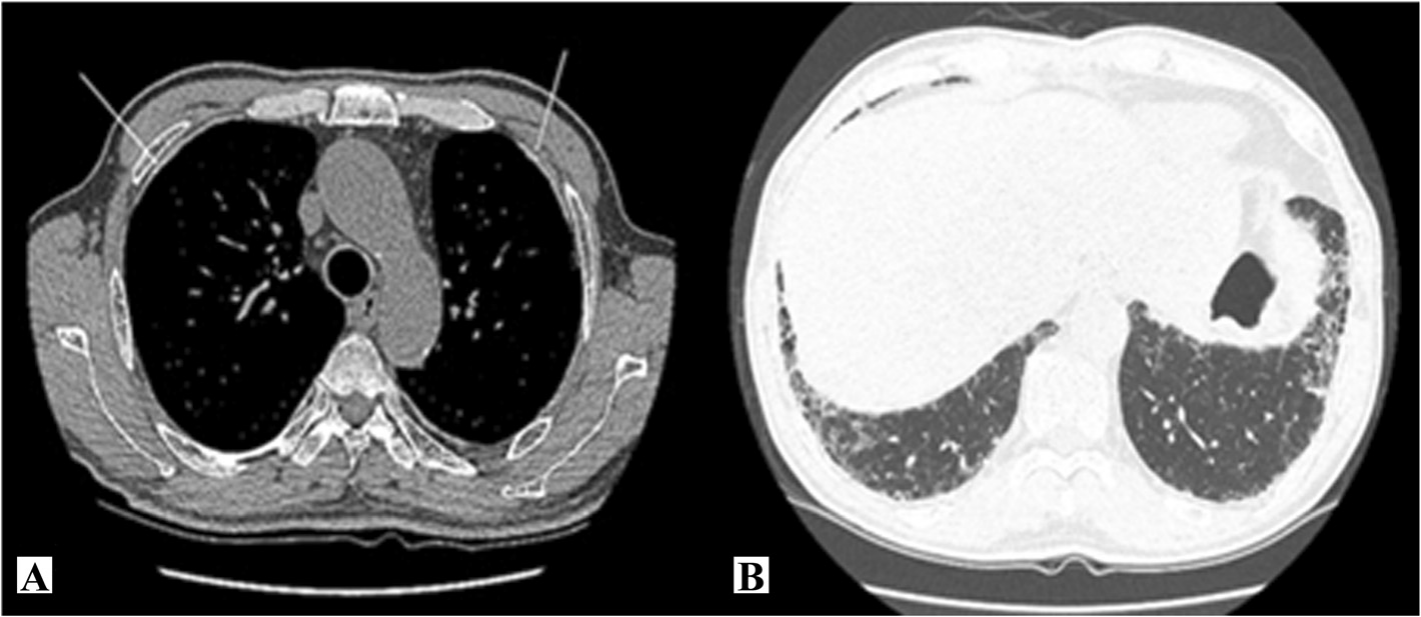
**Chest computed tomography images. A**. Discrete pleural thickening and calcification in the bilateral hemithorax. **B**. Fine reticular opacities, ground glass opacity, traction bronchiectasis, and honeycomb appearance in the bilateral lungs.

The chest CT images obtained on November 11, 2013 showed typical asbestosis findings of pleural thickening and calcification in both thoracopulmonary cavities, subpleural fine reticular opacity in the lungs, ground glass opacity, and honeycomb appearance (Figure 1). There were no significant findings from the chest X-ray images.

### Endoscopic findings

**Figure 2:**
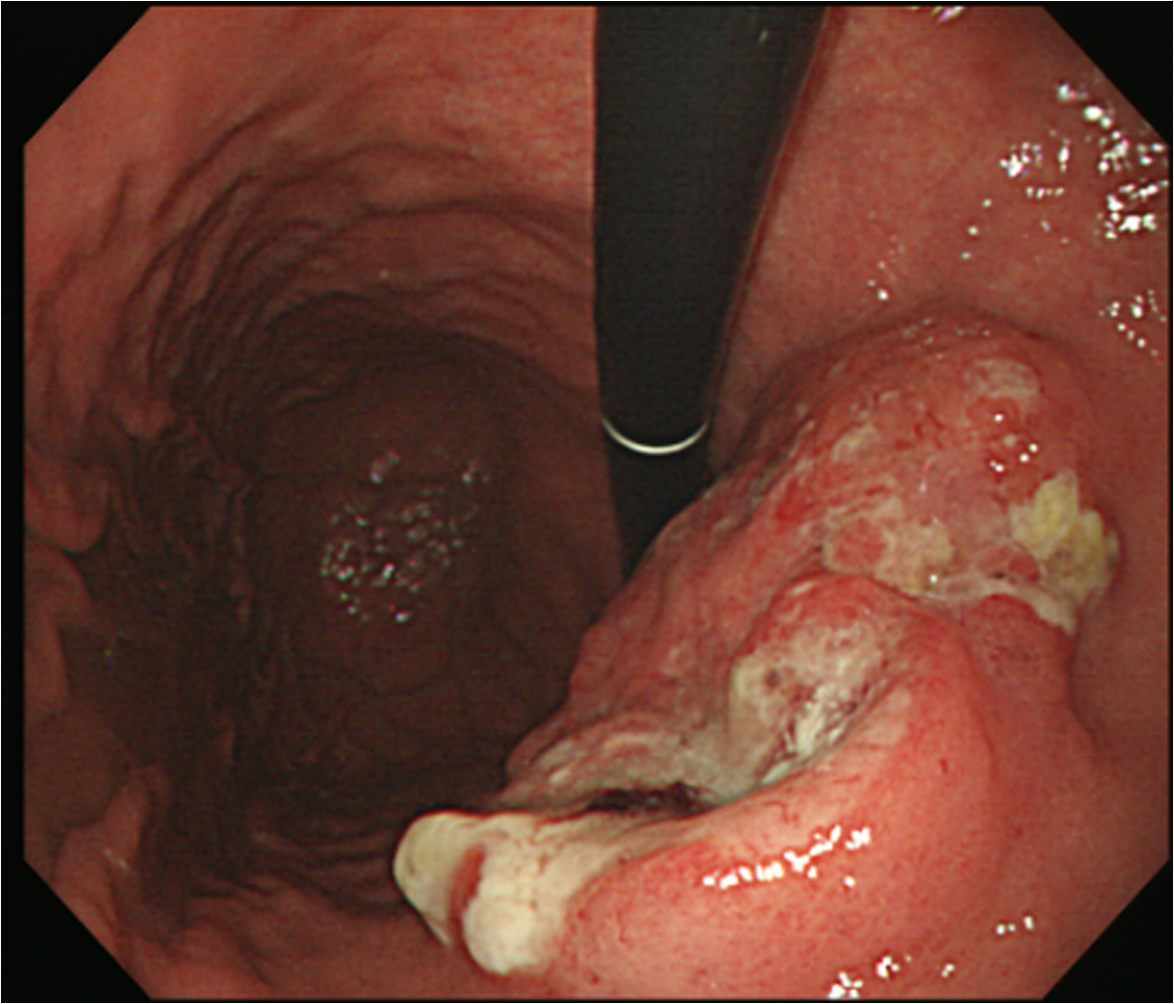
**The patient’s endoscopic examination revealed ulceration induced infiltrative changes and submucosal tumors, indicating advanced gastric cancer in the upper body lesser curvature anterior wall.**

In the upper gastrointestinal (GI) tract endoscopy conducted at a clinic in Pusan on July 3, 2010, reflux esophagitis and erosive gastritis were observed. Another upper GI tract endoscopy conducted on September 8, 2012 showed campylobacter-like organisms (CLOs) (+), suspected progressive gastric cancer, and atrophic gastritis (Figure 2).

### Pathologic findings

**Figure 3:**
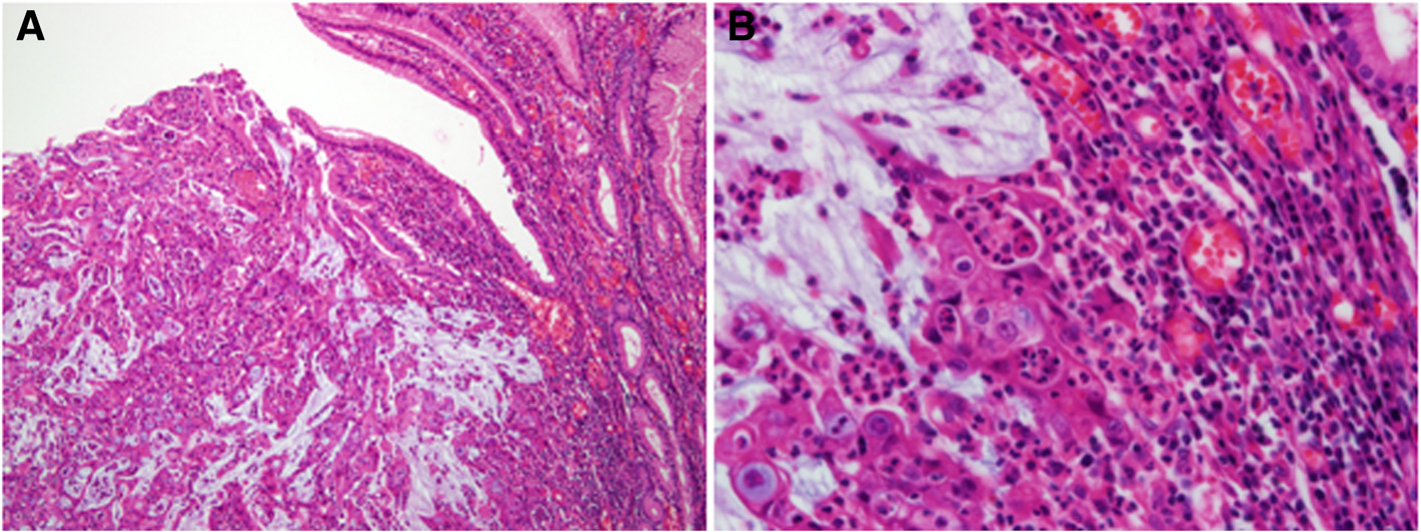
**Pathologic findings. A**. Adenocarcinoma without proper formation of gland structure, but with observablemucin production (hematoxylin and eosin staining, × 100). **B**. Enlarged nuclei with distinct nucleoli, and the presence of inflammatory cells surrounding tumor cells as compared to cells of a normal gland (hematoxylin and eosin staining, × 400).

The post-operative pathologic examination conducted on October 4, 2012 confirmed that the mass measuring 4.9 × 4.5 cm and located in the anterior wall of the stomach body was an adenocarcinoma (Figure 3).

### Assessment of asbestos exposure

#### Exposure to asbestos in textile factories

According to studies on asbestos levels in factories handling the chemical, the mean value before 1990 was 4.3 f/cc (standard deviation[SD] 4.3 f/cc), and that in 1990 was 2.3 f/cc (SD = 1.7 f/cc) [26]. The cumulated exposure of the patient in this case was calculated as follows:

$$ 2.3\ \mathrm{f}/\mathrm{cc} \times 12/8\ \mathrm{hours} \times 11\ \mathrm{years} \sim 4.3\ \mathrm{f}/\mathrm{cc} \times 12/8\ \mathrm{hours} \times 11\ \mathrm{years} $$

Thus, his level of cumulated exposure in the patient was estimated to be 38.0-71.0 f-yr/cc. According to the Helsinki criteria for asbestos-related diseases, the relative risk of developing lung cancer more than doubles when the cumulated exposure to asbestos is 25 f-yr/cc or higher [27].

Considering the patient’s work experience with chrysotile in the textile factory and his past working environment, he was thought to have been exposed to high level of asbestos.

#### Gastric cancer risk assessment of asbestos fiber exposure

Asbestos fiber exposure is reportedly associated with gastric cancer. In this case, the patient worked for 11 years weaving and spinning asbestos. Therefore, the possibility of gastric cancer caused by asbestos fiber exposure could not be completely excluded. In the meta-analysis of the association between asbestos fiber exposure and gastric cancer that compared any exposure versus none, the summary relative risk was calculated to be 1.17 (95% confidence interval [CI], 1.07–1.28). Similarly, in the comparison of high-dose asbestos fiber exposure versus no exposure, the summary relative risk was calculated to be 1.31–1.33 [28].

The standard mortality rate (SMR) of gastric cancer tends to increase when that of lung cancer does. Since the cumulative asbestos fiber exposure of this patient was 38.0–71.0 f-yr/cc, and his relative risk of developing lung cancer was more than two times, the SMR of gastric cancer was 1.34 (95% CI, 1.07–1.67) when that of lung cancer was > 2.00 [29]. When the SMR of lung cancer was >3.00, that of gastric cancer was 1.43 times higher [30].

#### Excess life cancer risk (ELCR) of lung cancer

The risk of asbestos fiber exposure was determined based on the incidence of lung cancer caused by such exposure and information obtained from the exposure evaluation. According to the United States Environmental Protection Agency (US EPA), there are three routes of asbestos fiber exposure: ingestion, respiration, and skin exposure [31]. However, asbestos is rarely absorbed through the skin, and no carcinogenic risk from ingestion has been reported [32]. Therefore, the ELCR from respiration was calculated to evaluate the health risk from asbestos exposure using the following equation [33].

$$ \mathrm{ELCR} = \mathrm{Exposure}\ \mathrm{P}\mathrm{oint}\ \mathrm{C}\mathrm{oncentration}\ \left(\mathrm{E}\mathrm{P}\mathrm{C}\right) \times \mathrm{Time}\ \mathrm{W}\mathrm{eighting}\ \mathrm{F}\mathrm{actor}\ \left(\mathrm{T}\mathrm{W}\mathrm{F}\right) \times \mathrm{Inhalation}\ \mathrm{U}\mathrm{nit}\ \mathrm{R}\mathrm{isk}\ \left(\mathrm{I}\mathrm{U}\mathrm{R}\right) $$

$$ \mathrm{E}\mathrm{LCR} = \mathrm{E}\mathrm{P}\mathrm{C} \times \mathrm{T}\mathrm{W}\mathrm{F} \times \mathrm{I}\mathrm{U}\mathrm{R} = 4.3\ \mathrm{f}/\mathrm{cc} \times 24/8\ \mathrm{h} \times \mathrm{\frac{1}{2}}\ \mathrm{day} \times 260/365 \times 2.1 \times 1{0}^{-2} = 9,648 \times 1{0}^{-5}. $$

EPC was calculated as 4.3 f/cc × 24/8 h using the reported asbestos concentration in factories handling the chemical. Time weighted average in this case was calculated using asbestos fiber exposure time, considering that the patient worked for 12 hours per day and 5 days per week (12/24 h/day × 260/365 days/yr). During his 11 years of exposure, the IUR was 2.1 × 10^−2^.

The theoretically acceptable carcinogen risk that corresponds to the natural death rate is 1.0 × 10^−6^ (one out of one million) [34]. However, in cases of environmental carcinogens, the acceptable risk is practically one in 100,000, considering the profit/loss against investments and status of engineering and analytical technologies [35]. The World Health Organization also suggests that the ELCR of carcinogenic substances is 1.0 × 10^−5^ (one out of 100,000) [36]. The ELCR of lung cancer for this patient was 9,648 × 10^−5^, which translated to an excess cancer risk of 9,648 in 100,000.

## Conclusion

The association of gastric cancer and asbestos fiber exposure is well known as asbestos is classified as a member of IARC group 2A. The patient in our case had significant asbestos fiber exposure to induce asbestosis.

In cases of concurrent gastric cancer and asbestosis, it is important to evaluate the significance of work-related asbestos fiber exposure and estimate the risk involved. However, it is difficult to use asbestosis as a surrogate for gastric cancer caused by asbestos fiber exposure, and death from asbestosis cannot be clarified, making it challenging to calculate SMR [36]. Therefore, the ELCR of lung cancer, which was determined to be 9,648 × 10^−5^, was used. Since it is known that as the risk for lung cancer increases, that of gastric cancer also dose, the above-mentioned result provide an important evidence suggesting that the incidence of gastric cancer of this patient was related to his asbestos fiber exposure.

Although asbestos-related cases studies on the simultaneous development of gastric and lung cancer [21], or gastric cancer, lung cancer, and malignant mesothelioma, and on multiple other malignant diseases have been reported [22], the literature on concurrent benign diseases and gastric cancer is scarce. Thus, to the best of our knowledge, this study is the first to describe a case of benign asbestosis occurring simultaneously with asbestos-related gastric cancer.

In a cohort study the death rate from gastric cancer of workers exposed to asbestos in New York and New Jersey was three times higher than that of the control group, and a dose–response relationship between the duration of asbestos fiber exposure and mortality risk from gastric cancer was indicated. The SMR of the group with less than 20 years of asbestos fiber exposure was 1.00, whereas the corresponding values for workers with 20–35 years and ≥35 years of exposure were 4.00 (95% CI, 1.47–8.71) and 3.42 (95% CI, 1.82–5.85), respectively [23]. In contrast, no statistically significant dose–response relationship between asbestos fiber exposure and gastric cancer was found in a large-scale cohort study conducted in Canada and the US that targeted textile workers (SMR = 1.16, 95% CI, 0.92–1.78) [24]. Furthermore a cohort study of 10,918 miners in Quebec, Canada who were exposed to chrysotile reported an SMR of 1.24 (95% CI, 1.07–1.48) and a positive dose–response relationship between the cumulated asbestos fiber exposure and the incidence of gastric cancer [25]. Since findings from cohort studies on the relationship between asbestos fiber exposure and gastric cancer development [37,38] remain controversial, the IARC classifies asbestos as a group 2A carcinogen for gastric cancer.

Gastric cancer could develop from various causes. In this study, endoscopic findings included *H. pylori* infection at the diagnosis of gastric cancer. However, it was difficult to conclude that *H. pylori* caused gastric cancer in our patient because his previous endoscopic findings were normal. Test for CLOs was not conducted until the 2010 endoscopy, possibly because of no suspicious endoscopic findings. Therefore, despite the presence of *H. pylori* in endoscopic findings, the patient’s gastric cancer could be affected by not only *H. pylori* but also asbestos fiber exposure. Additionally, his medical history did not include other diseases such as gastric ulcer or atrophic gastritis that could lead to gastric cancer development. Although the patient had a history of smoking and alcohol consumption, previous studies did not clearly demonstrate the relationship between such habits and gastric cancer [39,40]. In the meta-analysis of the relationship between smoking and gastric cancer, the incidence rate of gastric cancer due to smoking is more than 1.53 times [41]. However, the disease risk decreases if one quits for 10 years [42]. The patient in this case reported a smoking history of 23packs per year, but quit in 1995.

If one breathes in asbestos fibers, most of them are removed from the lungs in a layer of mucus to the throat and subsequently swallowed into the stomach. Alternatively, if one swallows asbestos fibers, either those in drinking water or being removed from the lungs to the throat, almost all of the fibers are excreted through the digestive system within a few days. A small amount of fibers may penetrate into the cells that line the stomach or intestines, and eventually enter the blood stream. Some of these become trapped in other tissues, whereas other are secreted in the urine [43]. The accumulation of ingested asbestos fibers might cause cancer. The patient in this case used to drink water and eat snacks while working at the textile factory. Thus, he might have ingested a large amount of asbestos fibers. Asbestos ingestion via food intake [44] or transport of the chemical to the stomach via respiration [45] has been suggested to cause gastric cancer. It has also been reported that gastric cancer development associated with chrysotile is more frequent than that with crocidolite [46]. Although the frequency of GI tract diseases caused by asbestos is lower than that of the lungs and peritoneum, the chemical is closely associated with gastric cancer.

When a patient is exposed to various environmental and occupational risk factors for gastric cancer, OEM physicians must carefully consider the effects of each factor. In this case, the patient’s gastric cancer could have been easily associated with *H. pylori* infection. Although *H. pylori* evidently cause gastric cancer, the infection period of our patient was short and the development of other diseases such as atrophic gastritis and gastric ulcer was not reported. On the other hand, his exposure to asbestos fibers was significant and the latent period was long enough for gastric cancer development.

A limitation of this study was that the risk of gastric cancer was indirectly inferred using the ELCR of lung cancer rather than, directly calculated based on levels of asbestos fiber exposure. Furthermore, the ELCR was calculated using the IUR reported by the USEPA in this study, which might be different than that in Korea. In addition, the extrapolation of epidemiological study into individual patient’s risk assessment was another limitation of our study.

The patient in this case suffered from concurrent asbestosis and gastric cancer. His risk of gastric cancer from asbestos fiber exposure was evaluated via exposure estimation and risk assessment.

A method to estimate the risk of gastric cancer from asbestos exposure with a combination of general and occupational factors might also be needed from the OEM perspective.

## Consent

Written informed consent was obtained from the patient for publication of this Case report and any accompanying images. A copy of the written consent is available for review by the Editor-in-Chief of this journal.
